# Understanding the Spectrum of Anxiety Responses to Climate Change: A Systematic Review of the Qualitative Literature

**DOI:** 10.3390/ijerph19020990

**Published:** 2022-01-16

**Authors:** Catriona Soutar, Anne P. F. Wand

**Affiliations:** 1 Sydney Local Health District, Sydney 2050, Australia; 2 Specialty of Psychiatry, Faculty of Medicine and Health, University of Sydney, Sydney 2050, Australia; anne.wand@sydney.edu.au; 3 School of Psychiatry, Faculty of Medicine, University of New South Wales, Sydney 2033, Australia

**Keywords:** anxiety, climate change, global warming, qualitative measures, eco-anxiety

## Abstract

Background: Knowledge about climate change may produce anxiety, but the concept of climate change anxiety is poorly understood. The primary aim of this study was to systematically review the qualitative literature regarding the scope of anxiety responses to climate change. The secondary aim was to investigate the sociodemographic and geographical factors which influence experiences of climate change anxiety. Methods: A systematic review of empirical qualitative studies was undertaken, examining the scope of climate change anxiety by searching five databases. Studies were critically appraised for quality. Content analysis was used to identify themes. Results: Fifteen studies met the inclusion criteria. The content analysis was organised into two overarching themes. The scope of anxiety included worry about threats to livelihood, worry for future generations, worry about apocalyptic futures, anxiety at the lack of response to climate change, and competing worries. Themes pertaining to responses to climate change anxiety included symptoms of anxiety, feeling helpless and disempowered, and ways of managing climate change anxiety. Relatively few studies were identified, with limited geographical diversity amongst the populations studied. Conclusions: The review furthers understanding of the concept of climate change anxiety and responses to it, highlighting the need for high-quality psychiatric research exploring its clinical significance and potential interventions.

## 1. Introduction

Climate change has been identified as one of the most pressing issues of our time, with major impacts on both physical and mental health [1]. Beyond the direct challenges that climate change poses to the determinants of mental health, such as threats to accessing basic needs (such as water, fresh air, food, and stable housing) and the trauma associated with extreme weather events, the broader psychological and emotional effects of climate change are increasingly being recognised [2,3,4]. This emerging field of study includes the interrelated phenomena of solastalgia, eco-anxiety, and ecological grief. Solastalgia, or the distress produced by the degradation of one’s home environment, was a concept first introduced by Albrecht [5], and it has been the subject of a growing body of literature since [6,7]. Ecological grief is a related concept, relating to grief experienced at ecological losses, i.e., to the loss of species, ecosystems, and meaningful landscapes [8,9]. Eco-anxiety has been defined by the American Psychological Association as “a chronic fear of environmental doom” [10].

Climate change anxiety has been proposed as the most recognised form of eco-anxiety [11]. It has been defined as “anxiety associated with perceptions about climate change”(p. 2) [12], which may involve cognitive, emotional, or functional impairment and somatic arousal (bodily symptoms) [13]. A related concept, climate change worry, involves negative or apprehensive thoughts about climate change that may be repetitive, difficult to control, or persistent [14]. Thus, worry may be a component of climate change anxiety. In recent years, climate change anxiety has become topical within mainstream media [15,16]. Climate change was the leading worry for Australians in a recent national media survey [17], and up to 80% of young people report being somewhat or very anxious about climate change [18]. Climate change anxiety has also been well recognised within the academic literature [11,12] and formal studies have found a similar prevalence of anxiety as the media surveys [19]. Despite increasing recognition, climate change anxiety remains an emerging concept that is in the early stages of being understood [12]. To date, we are not aware of any studies that have systematically examined the breadth of experiences comprising climate change anxiety, which is essential to furthering our understanding of this concept. 

The aim of this systematic review is to explore the empirical qualitative literature examining the scope of climate change anxiety and the spectrum of responses to it. A secondary aim is to explore the factors influencing climate change anxiety. Qualitative methodologies are most appropriate to develop an in-depth understanding of a new phenomenon, as they explore subjective experiences and narratives in an open way [20]. Thus, this review focuses on studies using qualitative approaches in order to richly describe the nature and scope of anxiety in response to climate change.

## 2. Materials and Methods

### 2.1. Search Strategy

The review was conducted according to PRISMA reporting guidelines. A search of the published literature was performed using the databases Medline, PsychINFO, ProQuest, Web of Science, and Scopus. Search terms used were ‘climate change’ OR ‘global warming’ combined with ‘anxiety’ OR ‘worry’ OR ‘fear’ OR ‘eco-anxiety’. MESH Headings were utilised where possible. The search was limited to English-language papers utilising qualitative methodology to examine the psychological/emotional experiences of, and responses to, climate change that fell within the anxiety spectrum. Only empirical research published in peer-reviewed journals from January 2000 to August 22nd, 2020, was included. Duplicate citations were removed after the initial search. 

The results of the database searches were initially screened by title and abstract by the first author. All articles potentially meeting the inclusion criteria (described below) were reviewed in full text. Where it was unclear whether an article met the inclusion criteria, it was discussed with the co-author until a consensus was reached. 

To maximise the comprehensiveness of the search, citation chaining was performed on all papers that met the inclusion criteria to identify further relevant papers. In addition, three experts in the field of climate change and mental health were contacted for further recommendations. 

### 2.2. Inclusion and Exclusion Criteria

Any studies that documented anxiety-related emotional or psychological experiences (e.g., anxiety, fear, worry) in relation to climate change using qualitative methodology (including mixed methods) were eligible for inclusion. Papers were included regardless of whether anxiety about climate change was the primary or secondary focus of the study, or an emergent finding. Studies of climate change worry, a component of climate change anxiety, were eligible for inclusion if they examined the nature and content of anxious cognitions about climate change. 

Papers that examined anxiety responses to individual environmental phenomena (e.g., natural disasters, sea level rises, or environmental change within a specific landscape) were excluded. Although related, they are considered to be different phenomena to anxiety in response to the knowledge of climate change, which was the focus of this study. Papers studying perceptions of climate change that did not identify anxiety responses were also excluded, as were papers reporting only quantitative data. Opinion pieces, reviews, and grey literature were excluded from the review. 

### 2.3. Quality Rating

Each paper that met the inclusion criteria was assigned a quality rating as per the guideline checklist developed by Attree and Milton [21]. This guideline describes a systematic rating scale for qualitative research papers. Each study is rated on criteria that include research background, aims and objectives, context, appropriateness of design, sampling, data collection and analysis, reflexivity, value of the research, and ethics. A total score is assigned based on the grade for the majority of sections. Studies receiving ‘A’ scores have no or few flaws, ‘B’ have some flaws, ‘C’ have considerable flaws but are still of some value, and ‘D’ scores have significant flaws that threaten the validity of the study as a whole.

Each paper was analysed and scored independently by both authors. Where assigned scores differed, there was discussion of ratings until a consensus was reached. As per Attree and Milton’s recommendations, papers with a score of D were excluded from the analysis [21]. 

### 2.4. Data Extraction and Thematic Analysis

Data were extracted independently by both authors. The standardised form for data extraction included study details (author, year, country of study), aim, design and methodology, participants and setting, anxiety themes identified, other themes identified, factors influencing the emotional or psychological experience of climate change, and quality rating, with comments on methodological factors. 

A thematic analysis was conducted by the lead author. The methodology followed the six phases described by Braun and Clarke [22], using a process of inductive analysis. Using this technique, data are coded and themes are derived directly from the data presented, without pre-conceived themes and with a broad research question [22]. The phases included familiarisation with the data by reading and re-reading the texts, generating initial codes, searching for themes, reviewing themes, defining and naming themes, and producing the report. Emergent themes were discussed with the co-author until a consensus was reached. Themes were revised iteratively in light of new data until the final themes and sub-themes were established. 

## 3. Results

**Figure 1:**
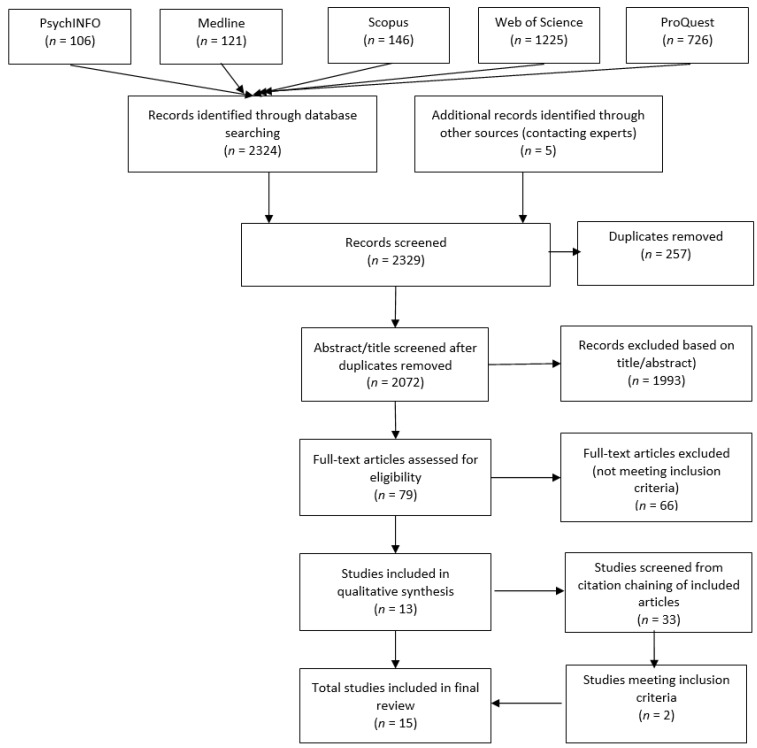
PRISMA flow diagram.

The PRISMA diagram outlines the search process and selection of papers (see Figure 1). 

### 3.1. Study Characteristics

A total of 15 studies met the inclusion criteria [23,24,25,26,27,28,29,30,31,32,33,34,35,36,37]. The study information and detailed findings are presented in Table 1. Four studies were conducted in North America [24,26,29,30], three in Australia [25,33,35], two in Norway [31,34], and one each from South Korea [23], Sweden [37], Ghana [32], and Tuvalu [27]. One study was conducted across four nations (Fiji, Cyprus, New Zealand, and the United Kingdom) [36] and one study did not identify its origin [28]. The majority of studies (10/15) were published in the previous five years. All but one [37] focused on adults, though the two Norwegian studies included a small number of teenagers within groups of predominantly adult participants [31,34]. 

Six of the papers were purely qualitative and the other nine used mixed methods (see Table 1). The stated goals of the studies were variable; nine sought to study mental wellbeing and emotions in the context of climate change [24,27,28,29,30,31,35,36,37], and six explored broader perceptions of climate change [23,25,26,32,33,34]. Two studies included quantitative measures of distress and anxiety [27,29]. Five studies predominantly investigated for, or identified, climate change anxiety [23,27,28,29,35], whereas ten studies focused on responses more consistent with climate change worry [24,25,26,30,31,32,33,34,36,37]. 

Five studies scored the highest quality rating of A, five were rated B and four were rated C (see Table 1). A single study [31] was rated D and was thus excluded from the analysis. 

### 3.2. Thematic Analysis

The results of the systematic review were divided into two broad areas of understanding about climate change anxiety; namely, (i) the “scope of climate change anxiety”, i.e., what was the specific reason for or focus of anxiety, and (ii) “responses to climate change anxiety”, which included emotional responses and ways of coping with anxiety (Table 1). Multiple themes emerged for each and are presented below. Results for the secondary aim, to explore the factors influencing climate change anxiety, are also provided in Table 1.

#### 3.2.1. Themes Related to the Scope of Climate Change Anxiety

A number of key themes emerged regarding the focus of climate change anxiety, which included worry about threats to livelihood, worry for future generations, worry about apocalyptic futures, anxiety at the perceived lack of response to climate change, and competing worries. 

##### Worry about Threats to Livelihood

Disruptions to livelihood were a source of anxiety in many studies [24,26,27,29,30,36]. Threats to daily life identified by participants included adverse economic changes, migration, extreme weather events, and lack of disaster preparedness. Many feared the loss of access to resources resulting in water scarcity, disruptions to food supplies and crop production, and impacts on local agriculture and the economy. Villagers in Fiji were concerned that:

“*Maybe in the future our plants can’t grow and we have to go buy them from the market*”

[36] (p. 105)

Water scarcity was a concern in a lakeside community in Canada:

“*You need water, like you can’t survive without that so I do worry about…access to clean water*”

[26] (p. 78)

Financial worries were the most prominent for the predominantly male farmers interviewed in America:

“*as with most, my worries generally stem from financial stress*”

[29] (p. 91)

Several studies highlighted concern about climate change-related migration. This included people who anticipate being forced to migrate:

“*I know I’ll be leaving soon, but when news comes that Tuvalu is affected or will sink, it makes me cry. Because I was born here, I’m Tuvaluan*”

[27] (p. 4)

In contrast, in countries less immediately threatened by climate change, such as the United Kingdom, the worry related to how to accommodate incoming climate refugees [36]. 

The increased frequency of extreme weather events and their links to livelihoods was a concern in Wisconsin:

“*I’m afraid we’re really gonna start getting hit with like massive tornadoes. That’s my biggest fear*”

[30] (p. 6)

There were divergent subthemes of “opportunity” and “already adapted”. Some perceived new livelihood opportunities related to the changing climate. A respondent in the fishing village of Kodiak, Alaska, was optimistic:

“*A disaster for some may mean prosperity for others – as polar ice melts, we might benefit as more shipping traffic comes through this area*”

[24] (p. 292)

The business opportunities presented by the challenges of climate change were emphasised by sustainability consultants in corporations in Australia:

“*Answering the challenge and being part of the climate change solution can have a multitude of immediate and long-term benefits for business*”

[35] (p. 1572)

Some people did not perceive threats to their way of life, as they had “already adapted”. For example, among farmers in Ghana, there was a degree of confidence about adapting to environmental change, which seemed to mitigate against anxiety:

“*Let’s face it, people have already seen extreme weather events in the past. Very bad ones. So they keep finding innovative ways of dealing with the weather changes. That’s why we are aware but don’t generally worry about environmental changes*”

[32] (p. 52)

##### Worry for Future Generations

Worry about future generations was a prominent theme in more developed countries [24,26,29,34,36]. The uncertainty of what sort of world would be left for children and grandchildren was a source of distress. For example, a participant in Ontario, Canada, stated:

“*We are actually taking our planet on a crash course and as a grandmother, I am deeply, deeply, deeply concerned about this. I have a one-year-old granddaughter now. You know we are leaving nothing for her*”

[26] (p. 80)

A divergent subtheme regarding worry for future generations was that of hope. Future generations were seen as a source of hope and potential solutions amongst young people in South Korea:

“*Hope may come from education*”

[23] (p. 67)

##### Worry about Apocalyptic Futures

For some, climate change was a “frightening scenario” [34] (p. 784) that evoked anxiety. “Catastrophe” and “apocalypse” were amongst the single words chosen to describe how participants felt about climate change in an American study [30] (p. 4). Students in South Korea associated climate change with doomsday scenarios they had seen in the media:

“*Something like this will happen to humans; we cannot prevent destroying the earth…we will die*”

[23] (p. 68)

##### Anxiety at the Perceived Lack of Response to Climate Change

The failure of others to take action was a source of anxiety. Participants felt that climate change could only be talked about in certain groups, which increased their anxiety [23,30,35], and not everyone shared the participants’ worries:

“*The first thing I associate when I hear climate change is terrified, you know. People don’t think it exists, or it’s happening, or nobody is going to do anything about it*”

[30] (p. 5)

##### Gaining Perspective—Competing Worries

Some participants struggled to place climate change amongst multiple other more pressing concerns in their daily life [30,32,33,34]. This was true in Western cultures, such as Wisconsin, USA:

“*There’s so much to be worried about, it’s diluted you know, what we have time to talk about*”

[30] (p. 5)

However, the awareness of competing worries was particularly true of participants in areas where resources were already scarce. Grape growers in Australia identified multiple financial and political stressors that they placed ahead of climate change:

“*Climate change is the least of my worries*”

[25] (p. 6)

Amongst Yolgnu people in Arnhem Land, Australia, local social and ecological issues, such as mining and observed seasonal changes, were highlighted as more immediate concerns than anthropogenic climate change which, in fact, was barely mentioned [33]. Climate change was not viewed as a separate entity from other worries, but was perceived as interwoven and likely to exacerbate existing worries, which, as in Ghana [32], were deemed to be much more important than climate change.

#### 3.2.2. Themes in Responses to Climate Change Anxiety

A spectrum of responses to climate change anxiety emerged, including symptoms of anxiety, the feelings of helplessness and disempowerment that climate change anxiety evoked, and finally a complex theme encompassing the ways of managing climate change anxiety. Distancing and avoidance, taking action, fostering support, adapting, and actively choosing optimism and hope were themes regarding strategies employed to manage climate change anxiety.

##### Symptoms of Anxiety

Participants described worry about climate change in ways that were consistent with symptoms of clinical anxiety. This included feelings of suffocation [23], panic amongst climate change activists [28], and rumination on negative emotions of guilt and worry [37]. In Tuvalu, residents described disturbed sleep, poor concentration, inability to relax, and other effects on function related to anxiety about climate change:

“*Sometimes I want to sleep, but I can’t because those thoughts about climate change keep popping up…thoughts about this distract me from my study*”

[27] (p. 4)

##### Feeling Helpless and Disempowered 

People across Europe [34,36], North America [24,30], Australia [25], and in Tuvalu [27] described a sense of powerlessness and feeling overwhelmed. They did not know what to do to tackle the problem of climate change, feeling individual measures were futile, which led to feelings of depression and anxiety:

“*I feel that you resign a little, this is too big. This makes you feel like: Help, what can you do other than trouble yourself?*”

[34] (p. 785)

Disempowerment was a strong theme amongst South Australian grape growers, where there was distrust of climate change information because:

“*some of the information is too extreme, so if those projections happen we’re finished anyway*”

[25] (p. 7)

##### Managing Climate Change Anxiety

Five themes emerged describing strategies to manage climate change anxiety.

- Distancing and avoidance

A key theme which emerged as a way to manage climate change anxiety was distancing and avoidance. Some saw climate change as something geographically and temporally distant, which was protective against anxiety [24]. Some downplayed the seriousness of climate change to alleviate worry, such as this university student in Norway:

“*I do not think it [global warming] is as human-made…as it allegedly is, according to the tabloids…I do not think it is as bad as they want to describe it*”

[34] (p. 789)

Even those who engage with climate change on a daily basis in their work made conscious decisions to distance themselves from climate change anxiety. For activists, this was a key step in resolving feelings of crisis brought on by knowledge of climate change:

“*I barely think about climate change now. It’s in the background of my life all the time, but I rarely sit and actually talk about climate change or read very much about it*”

[28] (p. 230)

Climate scientists and sustainability managers both emphasised rationality in their daily work, managing emotions by restricting or compartmentalising. However, this approach to coping related to emotions in general and not anxiety specifically:

“*I think a lot of scientists convey the impression that they have no feelings at all about these issues*”

[28] (p. 236)

b. Taking action

A move towards action was another strategy that was used to manage worry about climate change. Young people in Sweden took actions, such as researching solutions and modifying behaviour, to cope with climate change anxiety [37]. Amongst farmers in the USA, “taking action” was linked to a sense of resilience:

“*Action, not worry, solves the problem*”

[29] (p. 91)

This sentiment was echoed by climate activists who developed a sense of agency that allowed them to move forward:

“*Action is the antidote to despair*”

[28] (p. 229)

c. Fostering support

Actively seeking support was an important theme that emerged in young people [37] and in the specific population of climate change activists who were dealing with overwhelming feelings about climate change [28]. The activists consciously held positive ideas about the future and identified the importance of a network of practice and culture of self-care:

“*We build into it after the event, doing something where we talk about the emotions of how to deal with that*”

[28] (p. 234)

d. Adapting

Developing confidence in the ability to adapt to climate change appeared to be linked to a sense of resilience. This was seen in general community members in America:

“*There are so many things we don’t know. We adapt. We’ve always adapted*”

[24] (p. 291)

Confidence about adaptation was particularly found in groups of farmers in the USA [29] and in Ghana [32]. These farmers appeared to be accustomed to adaptation, and this flexibility allowed them to deal with depressed and anxious feelings:

“*I see the changes and how they are affecting my farm but I am changing how I am farming in response to climate change rather than being depressed by it*”

[29] (p. 91)

e. Optimism and hope

Actively choosing to be optimistic and hopeful were responses used to manage climate change anxiety [23,24,26,35,37]. Focus on positive emotions was notable amongst young people in Sweden [37]. Positive reappraisals of the problem, positive thinking, and fostering existential hope were subthemes identified in the Swedish youth:

“*You have to feel hope to make things any better. If no one felt hope then you might as well give up*”

[37] (p. 547)

Focus on solutions and trust in science, policy, and environmental groups, amongst others, also helped young people cope with anxiety:

“*Because a lot of people are working, planting new trees, dealing with the waste and exhaust fumes from cars*”

[37] (p. 549)

Hope was also harnessed by sustainability managers in their daily work in Australian corporate environments:

“*I guess I’ve always been a bit of an optimist and you have to be in this game. I’ve got a hope in terms of human ingenuity that we all trade out of this somehow*”

[35] (p. 1569)

#### 3.2.3. The Intersection of Anxiety, Sadness, and Solastalgia

Parallel to climate change anxiety, people described feelings of sadness and loss that were difficult to separate from their experiences of worry [23,24,27,30,36]. They repeatedly referred to local changes observed in their environment, which were seen as personal experiences of climate change. A loss of culture and community was a source of both anxiety and sadness in Fiji:

“*Seeing the changes makes me feel sad because people are not engaged…in helping protect the village and community*”

[36] (p. 105)

Changes in landscapes and seasons were referred to with a sense of loss in Korea:

“*Now I do not feel spring or fall weather as much now...I feel like I lost something*”

[23] (p. 68)

The emotional impact of local environmental change was strongly felt amongst Indigenous Yolgnu people in Arnhem Land, Australia, with their unique connection to the land:

“*What we are doing to mother nature. Mother nature is now weeping*”

[33] (p. 686)

## 4. Discussion

The aims of this systematic review were to qualitatively explore the scope of climate change anxiety as a concept and to investigate the spectrum of anxiety responses to climate change. A secondary aim was to explore the factors influencing climate change anxiety. This systematic review of the qualitative literature contributes to our understanding of this important, understudied topic. A qualitative approach is best suited to develop an in-depth understanding of new concepts, such as climate change anxiety [20]). 

The scope of anxiety about climate change was broad. The majority of studies explored aspects of climate change worry, a component of climate change anxiety. The review identified that “worry about threats to livelihood” was a major concern across all populations, with a breadth of focus from anxiety about access to food and water, to anxiety about finances, population movement, and natural disasters. It is surprising that no known previous study has explored the specific focus of climate change anxiety in a quantitative manner, despite numerous studies measuring the prevalence of climate change anxiety [19,38,39,40,41,42,43,44,45,46], and studies assessing the scope of environmental worries beyond climate change [13,47,48,49]. The theme of “worry about future generations” has been identified previously [50], as have correlations between parenthood and anxiety about climate change [41]. In this study, “worry about future generations” was more prominent amongst Western countries [24,26,29,34,36], where participants tended to perceive climate change as a future rather than current threat. Anxiety about “apocalyptic futures” is likely to be found in those with egalitarian beliefs, who have higher perceptions of catastrophe related to climate change [38].

The systematic review revealed patterns in the demographic characteristics of participants and climate change anxiety, addressing the secondary aim of this study. The key factors influencing emergent themes for the scope of climate change anxiety were vulnerability to climate change, socioeconomic factors, and occupation (Table 1). Four of the twelve studies included participants from either developing countries (Fiji, Ghana, Tuvalu) or disadvantaged socioeconomic groups (Arnhem Land, Australia). These participants are arguably amongst the most vulnerable to the effects of climate change [1]. Despite their high vulnerability, two of these studies identified a relative lack of anxiety or worry about climate change [32,33] when compared to more developed nations in North America and Europe [24,26,30,34]. There are several potential explanations for this finding. In Arnhem Land, it was noted that Western concepts of climate change were very poorly understood [33]. Further, more immediate concerns about day-to-day survival were prominent there and in Ghana, giving rise to the theme of “competing worries”. Amongst populations already struggling to survive on the land, climate change can be seen as “yet another stressor” [51] (p. 1). In Tuvalu, by contrast, knowledge of climate change is strong, exemplified by the commonly known trope that “Tuvalu is sinking” [27] (p. 2). Consistent with this, very high levels of climate change anxiety were found in Tuvaluan residents [27], reflecting the level of threat that climate change poses to this island nation [52].

Occupation emerged as a likely influence upon the scope of anxiety about climate change. In North America, farmers (in a predominantly white male participant group) identified financial concerns about the impact of climate change [29]. Grape growing farmers in Australia showed a comparative lack of worry about climate change, as they were more concerned with perceived immediate stressors, such as farming costs and viability, alongside scepticism about the existence or seriousness of climate change [25]. Other farming populations have been demonstrated to hold higher rates of scepticism, with associated lower rates of worry about climate change, than the general public [43,53]. The present review also found that farmers in Ghana have little anxiety about climate change, as they are confident in their ability to adapt [32].

A gender-related difference in the scope of anxiety might be expected, given that women often experience higher levels of climate change anxiety than men [54,55,56]. However, the impact of gender on the scope of anxiety was not well explored by the reviewed studies. Only one study noted that women were more likely to worry about future generations, while men worried more about finances [24]. It was not possible to identify any patterns relating to age and the scope of anxiety, as the only paper that specifically studied young people assessed only responses to climate change anxiety, not the scope of anxiety [50]. Given the disproportionate impact of climate change on young people, further study in this population is needed. 

The second group of themes that emerged in this review were the responses to climate change anxiety, which ranged from “symptoms of anxiety”, to “feeling helpless and disempowered”, and “managing climate change anxiety: distancing and avoidance”, “taking action”, “fostering support”, “adapting”, and “optimism and hope”. The proximity to the effects of climate change corresponded with anxiety responses. “Symptoms of anxiety”, and the subsequent impact on daily function, were described by Tuvaluan citizens [27], who arguably face the most tangible threat from climate change. Similarly, the proximity of climate change activists to the realities of climate change threats precipitated feelings of panic [28], whereas such intense experiences of anxiety were less evident in other populations studied within this review. Feeling “helpless and disempowered”, however, in the face of anxiety about climate change, was a phenomenon commonly expressed across diverse geographical locations [24,25,27,28,30,34,36]. It is likely that this emotion is influenced by individual ontological belief systems, which have been shown to mediate feelings of helplessness in association with climate change anxiety [38]. 

This review did not reveal any clear factors influencing the particular ways of managing climate change anxiety. Compared to factors influencing the scope of climate change anxiety, it is likely that responses to anxiety (including worry specifically) are less influenced by characteristics, such as gender, location, or social group, but by individual psychological factors and ontological beliefs [39,40,57]. Further, the significant heterogeneity of climate change understanding, perception, and engagement in populations across the world is an ongoing challenge for research [54,57]. “Fostering support” and “distancing and avoidance” have small but significant effects on lessening the experience of psychological distress related to climate change [58]. Among 12-year-olds and adolescents, higher levels of worry were found in those who used “problem-focused coping”, where one focuses on ways to solve the problem, such as searching for information [50,59]. It was suggested that 12-year-olds who emphasised the positive affects of optimism and hope may be buffered against negative affects, including worry about climate change [59]. The importance of hope is increasingly recognised within the climate change literature as a part of psychological adaptation and as a way of overcoming difficult emotions associated with knowledge of climate change [60,61].

### 4.1. Solastalgia and Sadness

In this review, solastalgia and sadness emerged as key affects linked to climate change anxiety. Whilst quantitative results demonstrate that anxiety and sadness are frequently the two most common emotional responses to climate change [24,27], the qualitative approach of this paper highlights how closely these emotional states are interconnected, just as in clinical populations [62]. The experience of solastalgia and its relevance amongst Indigenous peoples is particularly recognised [7,63,64] and was also found in this study [33].

### 4.2. Clinical Implications

This review highlights the spectrum of anxiety people may experience in relation to climate change, and their responses to those anxieties. Worry, a cognitive component of climate change anxiety, was most commonly explored in these studies. Clinical presentations related to climate change anxiety have not been well elucidated, although they have been recognised in psychotherapeutic spaces [65] and at an obsessive compulsive disorder treatment clinic [66]. The treatment of eco-anxiety, a closely related concept, is an emerging area of research [67]. Evidence of clinical symptoms of anxiety emerged as a small but important theme in this review of the qualitative literature. Quantitative studies in this area report mixed results [19,46,68,69,70], but convincing evidence of a link between climate change anxiety and clinical anxiety is currently lacking. A survey of primary care patients in America identified a link between climate change concern and dysphoria, but no link to anxiety [70]. Another survey of Australian university students and community members found a small association between climate change distress and depression, and a moderate correlation between climate change distress and future worry [19]. Perceived ecological stress predicted depressive symptoms in a survey of parents in America [69]. None of these studies could demonstrate causation in either direction. Other studies have found no significant correlation between worry about climate change or ecology and psychiatric morbidity from anxiety [46,68]. 

An alternative view is that climate change anxiety is not a clinical entity, but rather an existential one [11]. The argument for understanding climate change anxiety as an existential worry, rather than a pathological worry of the kind recognised by psychiatry, is reflected by the fact that only two of the fifteen papers were published in medical or psychological journals [27,29]; the remainder appeared in environmental or sociological journals. Further, concern exists about pathologizing climate change anxiety, which has been described as a proportionate emotional response to the current environmental situation [12,60]. However, the descriptions of anxiety symptoms, associated distress, and impacts on behaviours and functioning that emerged from this qualitative review should not be ignored. 

Further research is required to identify people who are vulnerable to experiencing climate change anxiety at a level of clinical significance and who may benefit from intervention. Validated screening tools, such as the Environmental Distress Scale [71] or the scale developed by Clayton and Karazsia [13], may be helpful. People who screen positive for climate change anxiety may benefit from intervention. However, there is a dearth of literature guiding approaches to its management [67]. A recent scoping review highlighted emerging approaches to treating eco-anxiety, including both individual and group work, predominantly underpinned by ecotherapy, psychoanalysis, and Jungian depth psychology [67]. Notably, only two of the reviewed studies empirically evaluated their psychotherapeutic approach, emphasizing the need for more study [67]. 

Not all studies of the perceptions of climate change have identified anxiety within the spectrum of responses [25,32,42]. Further study is required to determine the reasons behind this, be they methodological or cohort based. Other emotional responses to climate change, such as anger, guilt, and hope, are likely to have their own patterns and associations [72] and require further study to determine their clinical significance and how they intersect with climate change anxiety. 

### 4.3. Strengths and Limitations of the Included Papers

Few of the papers explored participants’ knowledge and understanding of climate change, which may affect the reliability of results—why worry about something that you do not believe exists? In the two papers that did measure belief in climate change, it was as low as 58% in one study population [25], and showed significant variation (55–88%) between subsets of participants in another [24]. Another study included expressions of scepticism about the seriousness of the climate change threat [34]. In the study by Petheram and colleagues, it became clear to researchers that there were significant misconceptions about notions of climate change, and participants’ worry increased as they were educated over the course of the study [33]. Multiple other studies have highlighted the poor knowledge and understanding of climate change concepts [44], particularly in the developing world [73,74,75,76]. Knowledge of climate change can also be mediated by factors, such as media exposure, which is positively related to the experience of climate change anxiety [77], though its influence was not captured in any of the papers in this review. 

The quality of the included studies must be considered. One third of the studies were rated A (no or few flaws), one third were rated as B (containing some flaws), and four of the fifteen were rated C (considerable flaws but still of some value). More weight should be placed on the results from studies of higher quality. Reflexivity and ethics were particularly poorly addressed by the studies, perhaps because many were conducted within ethnographic rather than clinical research frameworks. Additionally, several studies were limited by inadequate sampling [23,28,30,34,35] and a limited application of techniques for validating results [23,24,26,30,34,35,36,37].

### 4.4. Strengths and Limitations of the Review

Strengths of this review include a thorough literature search intended to capture any study that included qualitative data on climate change anxiety (including worry), even where this was not a goal of the original study. The heterogenous populations and varied aims and methodology of the included studies were well suited to an exploratory qualitative approach.

Regarding limitations, the findings of this review may not be generalisable given the relatively small number of studies and the importance of location and population to understanding concepts of climate change anxiety that emerged in the analysis. The exclusion of grey literature and other unpublished papers may have limited the results through publication bias [78]. The terms used to identify the spectrum of anxiety responses were not exhaustive; for example, the word “concern” was excluded from the literature search as it returned a prohibitive number of titles for review (over 25,000). Finally, the distinction between climate change anxiety and climate change worry was not always clearly delineated, both within the individual reviewed studies and consequently in the broader thematic analysis of the systematic review. This highlights the need for clarity and standardization of the concept of climate change anxiety. 

## 5. Conclusions

Climate change anxiety is becoming recognised as one of the mental health effects of climate change. This review has identified a broad scope of worries about climate change, and a diversity of responses to this anxiety. Characteristics, such as occupation, socio-economic status, and proximity to climate change, appear to be important influences on climate change anxiety and related responses. The review furthers our understanding of the concept of climate change anxiety and highlights the need for future studies of this phenomenon to be conducted by clinical researchers. There is a pressing need to better understand the psychological and functional effects of climate change anxiety and to examine interventions to promote resilience and reduce clinically significant distress.

## Figures and Tables

**Table 1:** Description of included studies, thematic analysis, and quality ratings.

Author, Year, Country of Study	Design and Methodology	Aim	Participants and Setting	Anxiety Themes Identified (Including Themes about Worry Where Relevant)	Other Themes Identified	Factors Influencing Experience of Climate Change	Quality Rating and Comments
Anghelcev et al. 2015 South Korea	Semi-structured interviews and photo elicitation Zaltman metaphor elicitation technique (ZMET) and narrative thematic analysis	Primary: to illustrate the applicability of using ZMET in social marketing communication Secondary: to explore how climate change is perceived by young members of the Korean public	*n* = 12 (M 6, F 6) Age range 20–28 South Korean college students Half undergraduate and half graduate	Affective distress (fear, nostalgia, sadness) was one of 3 “deep metaphors” found Fear: apocalyptic futures (doomsday scenarios promoted by media) Fear accompanied by anxiety symptoms (feeling of suffocation, inevitable destruction)	Sadness (loss of world as we know it, futility of individual action and inability to reverse climate change) Nostalgia (memories of idealised past) Pandora’s box -tragic endings (loss of biodiversity and human habitat) - human greed (corporate greed for profits, selfish pursuit of comfort and gratification) -hope (education as agent of positive change, stricter governmental control) Two-faced Janus - discursive ambivalence (dual standards of accountability, ambivalent media discourse) - functional duality (technology as cause and solution, home as space of consumption and mitigation)	Participants perceived climate change as something geographically or temporally distant (not a direct threat or happening to them)	B -No information on ethical considerations -Bias in sampling (personal solicitation) -Quotes not matched to demographic data -No respondent validation -No reflexivity or consideration of cultural factors
Du Bray et al. 2017 Fiji, Cyprus, New Zealand, United Kingdom	Open-ended individual ethnographic interviews Interviews autocoded for positive and negative emotion words (counting of intext references) The method of qualitative data analysis of interview texts was not described	“To understand how emotional responses to climate change are inequitably distributed across people living in island nations with varying climate change vulnerability” (p. 1)	*n* = 272 Residents in 4 island nations: Fiji (*n* = 68), Cyprus (*n* = 40), New Zealand (*n* = 86), United Kingdom (*n* = 78) Gender and ages not provided	Fiji: worry about land, people, community, plants not growing, loss of self-sufficiency and cultural traditions Cyprus: worry about the future, rise of sea level, water scarcity, how to adapt to change New Zealand: worry about friends and family, speed of change, rise of global disasters UK: worry about grandchildren, population changes (incoming refugees)	Fiji: happiness and sorrow, pride in cultural heritage, sad at loss of livelihood traditions, new opportunities with relocation of younger generation Cyprus: concern/frustration with water shortage, anger/helplessness. Sadness about rainfall changes, unable to trust local agriculture NZ: sad at ecological loss. Hope for future generations, optimism UK: neutral, felt would not be impacted, cannot control climate change, therefore must not become upset about it	Island nations are vulnerable to climate change to varying degrees (UK and NZ less vulnerable, more adaptive capacity, Cyrus and Fiji more vulnerable with less adaptive capacity)	C -Poor description of context -Insufficient demographic information on participants -Sampling strategy not documented -No justification of sample size/data saturation -Data recording method unclear -Qualitative data analysis not recorded -No validation of findings -No consideration of limitations -No reflexivity or ethics considered
Du Bray et al. 2017 USA	Participant observation and mixed-method survey (16 open ended interview questions and 21 survey questions) Counting and coding of emotion words (KWIC (keywords-in-context) approach) Themes emerged deductively from the pattern of questioning The method of analysis is unclear	Primary: to determine “how emotional responses to climate change vary across sites with different experiences and projected outcomes as a result of climate change” (p. 286) Secondary: to determine “whether men or women were more likely to express emotions across these three sites” (p. 286)	*n* = 103 (M 50, F 53) Ages not provided Residents of 3 US cities: Mobile, Alabama (31), Kodiak, Alaska (36), and Phoenix, Arizona (36)	Worry and sadness for future generations Worry about financial issues/loss of livelihood (access to resources) or being unable to give children same livelihood opportunities Concern about changes to landscape Lack of worry in those who believe that climate change does not affect current generation Site specific: Alabama—worry for others but more worried by other natural events (hurricanes, storm water runoff) which will be made worse with climate change. Uncertainty and adaptation Alaska—anxiety was not a prominent theme Arizona—less emotional about climate change overall. Concern for younger generation (not have same experience)	Alabama: Large number of respondents did not believe in anthropogenic climate change Participants felt more prepared, able to adapt than other sites Alaska: Many respondents believed in anthropogenic climate change, (31% did not) Participants more likely to express hope for future, optimism or feeling safe, though there are others who anticipate negative consequences Some anticipated positive changes Arizona: Participants were least likely to indicate emotional reaction to climate change Resignation, unconcerned, feeling climate change is inevitable	USA less immediately impacted by climate change Alabama: Susceptible to hurricanes, coastal vulnerability, fishing/agriculture reliance Alaska: Fishing/wilderness reliance, Indigenous way of life is vulnerable to climate change Arizona: Urbanised environment, buffered from local ecology Gender: women more likely than men to evoke “worry”. Men worried about financial survival and livelihood. Women worried about future generations	B -Sampling strategy poorly described -Data collection and recording unclear -Method of analysis (counting keywords) is not the most appropriate to answer the study question in depth -Few quotes, not matched to demographic data -No validation of findings -No discussion of reflexivity or limitations -Good demographic description -Content validity through pre-testing -Some triangulation in data collection (field notes, participant observation, interviews) -Trustworthiness through deliberate selection of similar and different views
Fleming et al 2015 Australia	Mixed-method telephone survey (open-ended and Likert-scaled responses) and literature review Qualitative analysis using constructivist interpretations of grounded theory; used NVivo9 software Quantitative analysis: scaled responses were incorporated into codes, categories, and themes	To examine “how grape growers in this region perceive and prioritise climate change adaptation as an issue for their industry”	*n* = 50 Gender and ages not provided Grape growers in South Australia 50/68 = 74% response rate	Grape growers who were sceptical about climate change did not feel it would bring risks or opportunities Those who were convinced of it perceived greater risks	58% were sceptical of anthropogenic climate change Focus of worry not related to climate change included themes of: Significant concerns -immediate stress (cash flow, lack of succession, dwindling communities with limited labour access, lack of transparency) -loss of enjoyment in lifestyle Perceptions of climate change: scepticism Coping with stress -committed farmer -exiting the industry -positive outliers	High degree of scepticism about climate change influenced perception of risks and tendency towards action	A -Good response rate -Quality content analysis -Ethics approval -Identification of outliers validates results -Data collection not well recorded -No data saturation -Limited demographic data provided -No reflexivity discussed
Galway 2019 Canada	Semi-structured walking interviews Thematic analysis	“(1) To examine how community members of Thunder Bay understand, and think about the issue of climate change; (2) To examine how community members of Thunder Bay perceive climate change impacts and action; and (3) To consider the role of place in relation to climate change perceptions in the context of Thunder Bay” (p. 69)	*n* = 18 (M 8, F 10) Ages 20s–70s Residents in a remote city in Northern Ontario, Canada	Fear and concern/worry were most commonly reported emotions Worry for children and future generations Worry about future access to water, food, forests	Climate change as complex and interconnected Causes of climate change (fossil fuel industry, industrialisation, capitalism, greed, etc.) Climate (in)justice and ethics (intergenerational, marginalised communities) Taking notice of changes in the weather, seasons and extreme events Anticipated future impacts on water, food and forests -Perceptions shaped by experiences on land and water -Transformation at a range of levels, by a range of actors is needed to address climate change Other emotions included hopeful, frustrated, sense of urgency, depressed, angry/upset, guilt, sad	The importance of place, connection to local ecology Local and regional settings and relationships to this land/water/outdoors Extreme flood 2012 repeatedly referenced by participants	B -No data saturation to justify sample size -No triangulation -Quotes not matched to demographic data -Only one person analysing data -No validation of findings -Little discussion of limitations or alternative explanations -No reflexivity -Themes not explicitly presented -Questions pilot tested for clarity
Gibson et al 2020 Tuvalu	Mixed methods: structured interview with open and closed questions Questionnaire: culturally adapted Hopkins Symptom Checklist-25 Quantitative analysis: descriptive, correlational, and between-group analysis Qualitative analysis: method poorly described	To determine if residents in Tuvalu report distress on account of both local and abstract climate change To examine the extent to which reported distress impacts on daily functioning	*n* = 100 (M 50, F 50) Ages 18–24 (23), 25–39 (26), 40–54 (25), 55+ (26) Community members living on Funafuti atoll, Tuvalu	Quantitative 76% reported worry about abstract climate stressors Of those with distress, 79% reported impairment in daily life and 28% reported extreme impairment Qualitative Worry about safety, lack of disaster preparedness, having nowhere to go Impact of worry on daily function: poor sleep due to climate change thoughts, not going out, disturbs leisure time	Sadness prominent: 79% for local aspects and 77% for abstract climate change Approx. 84% reported worry/anxiety in response to local climate change stressors, and 79% reported sadness about environmental impacts, loss of homes and decreasing capacity to grow crops Distress and anxiety related to local climate stressors were more common than to abstract climate change	Extremely vulnerable country which may become uninhabitable due to sea level rise Awareness of this reality is found throughout Tuvalu Poverty: those with more financial hardship reported greater distress Distress attributed to climate change (local and abstract) showed small–moderate correlations with psychological distress more broadly	B -No respondent validation -Little reflexivity -No clear methodology presented for qualitative analysis -Themes not explicitly presented -Questions were piloted locally, assessed for internal consistency
Hoggett and Randall 2018 Country not reported	In-depth qualitative interviews Thematic analysis	To understand how scientists and activists psychologically manage their work on climate change To examine what emotional resilience factors are present	*n* = 16 (climate scientists 6, climate activists 10) Ages not well reported (activists 20 s–50 s, scientists 1 young, 5 senior) Gender not reported	Activists: ‘Crisis’ stage of journey involved urgency, terror, anger, feeling overwhelmed, disempowerment, difficult to resolve Anxiety from engagement with direct action (police, law) Burnout/depression in 9/10 activists Scientists: Anxiety from burden of responsibility Distress at disagreement with colleagues, public perception of science, fear of speaking out, media attacks	Activists’ trajectory: epiphany, immersion, crisis, resolution through sense of agency, action as antidote to despair Activists managing emotional impacts through positive and concrete view of the future, sophisticated and supportive network of practice, and emphasis on self-care Scientists’ trajectory: gradual realisation More variability in how knowledge affects private life Frustration/anger at public indifference Scientists managing emotional impacts through use of institutional defences: scientific progress, excitement of the work, detachment, rationality, specialisation, overwork	Both parties work directly with climate change issues in daily work Role (activist vs. scientist) demonstrated significant differences in trajectory of emotional impact, engagement in public sphere and managing emotional impacts	B -No funding source or consideration of ethics -Poor description of demographics of participants -Sampling bias -No data saturation -Data collection and recording unclear -Unclear how many people analysed the results -Quotes not matched to demographics -Limitations not discussed in detail -No reflexivity -Utilised member checking
Howard et al 2020 USA	Mixed method: quantitative survey with single open-ended question on how climate change was contributing to levels of mental distress Quantitative analysis: descriptive and correlational statistics, ANOVA Qualitative analysis: content analysis with coding methodology	Primary: “to examine the association between climate change risk perception and mental well-being among farmers and ranchers in Montana” (p. 88) Secondary: “to examine how climate change may be affecting the mental well-being of farmers and ranchers in the state” (p. 88)	*n* = 125 Gender and race not recorded but were predominantly male and white Age 18–34 (21.7%), 35–54 (49.2%), 55+ (29.2%) Farmers and ranchers in Montana, USA	Financial concern: worry about reduced crop yields, no funds to mitigate impacts, land and investments rendered useless, no one to buy business, children will not take over Operational planning: worry about unpredictability of climate affecting planting, crop choice	Resilience: changing farming, action response, have to adapt, be creative, flexibility, support politicians/groups helping	Agricultural workers with livelihoods depending on land Affiliation: organic farmers had significantly greater anxiety compared to conventional Operation focus: fruit/ vegetable farmers had significantly greater anxiety than grain/legume farmers Contribution to income: those with farming contributing to 10–70% income had significantly greater anxiety compared to 70–100% No significant differences in anxiety by age, generation or years working in agriculture	A -Possible sampling bias -Quotes not matched to demographic data -Very small but targeted qualitative component -Survey pre-tested for validity -Qualitative coding by two separate authors with high Cohen’s kappa agreement score
Kemkes and Akerman 2019 USA	Structured narrative interviews Interpretive phenomenological analysis	Explored participant understanding of climate change, worry about future changes, and who is responsible for addressing climate change	*n* = 17 (M 10, F 7) Ages 36–80 (mean age 56) Community members living on shore of Lake Superior, Wisconsin USA	Themes were not explicitly identified but extrapolated from data provided: Anxiety from failure of collective action and futility of individual change Uncertainty about local environmental changes, fear of major weather events Overwhelmed but acting ethically at an individual level “One-word responses” within the anxiety spectrum (scared, terrified, concerning, concerned)	Silence around climate change: inability to talk of climate change in certain settings Issue diluted amongst other things to be worried about Other one-word responses included negative (catastrophe, apocalyptic, hopeless/helpless, depressed), positive (hopeful, optimistic) words, as well as words related to scale of climate change (impending, inevitable, humbled)	Although demographic characteristics were gathered, they were not linked to themes derived from the data	C -Sample size and data saturation not addressed -Sampling bias -Themes not well elicited -No validation of findings -No discussion of limitations -No reflexivity, no consideration of ethics -Both authors performed data analysis independently
Norgaard 2006 Norway	Ethnographic data (field research), interviews, media analysis, participant observation, focus groups Analytic method not documented	Aim not clearly expressed To explore why people were not more actively engaged with global warming, with a focus on emotion and emotion management	*n* = 46 (M 25, F 21) Ages <20 (7), 20–35 (8), 35–60 (19), 69+ (11) Total 45 Range “19 to early 70 s” Residents in a rural Norwegian community	Fear related to loss of ontological security Fear of “being a bad person”, which was a threat to individual and national self-concept Unpleasant emotions (including fear) managed through selective attention (controlling exposure to information, not thinking too far ahead, focusing on something individual can do) which led to “movement non-participation”	Helplessness Need to maintain optimism, stoicism Observed changes over a lifetime Guilt of contributing to the problem Guilt and threats to identity managed by perspectival selectivity	Salience and visibility of climate change due to local changes Norway is a wealthy nation which benefits from oil production Occupation: activists and educators limit access to information to avoid being overwhelmed and enable them to continue their work	D -Unclear aims and title -Sampling not described -No data saturation -Poor description of data recording -No qualitative analysis documented -No respondent validation or independent data analysis -No consideration of limitations -Limited reflexivity, significant subjectivity -No use of empirical data to support claims -Funding source unclear -Issues with sampling, analysis, reflexivity, and ethics threaten overall validity
Nyantakyi-Frimpong and Bezner-Kerr 2015 Ghana	Ethnographic fieldwork guided by feminist political ecology theory Multimethod triangulation of focus groups, individual interviews, participant observation, meteorological data, household surveys Quantitative analysis: descriptive statistics (two sample test of proportions in SPSS version 21) Qualitative analysis: hand coding and analysis for themes; participatory ranking and scoring data analysed using a method by Tschakert to calculate incidence, severity and importance indices for each factor	“To explore the relative importance of climate change in the context of multiples stressors in semi-arid Ghana” (p. 1) “(i) What factors do farmers identify as most relevant for climate change resilience and adaptation, and how do these factors differ by gender, age and kinship relations? (ii) how important is climate change as compared to other factors that shape smallholder farming and food security?” (p. 40)	*n* = 135 8 Focus groups, *n* = 75 (young M 19, young F 21, elderly M 18, elderly F 17) In-depth interviews *n* = 60 (M 26, F 34) 6 were key informants (3 agricultural extension officers, 1 NGO worker, 1 nutritionist, and 1 health surveillance assistant). All were residents in 2 villages in northwest Ghana	Farmers are aware but do not worry about climate variability and change, compared to other concerns Farmers are used to extreme weather events and report that they already manage risk with adaptable farming systems and are used to innovating	Farmers perceive a change in climate accurately Concerns vary with gender - men worry about local weather events, food prices -women’s greatest concern is access to household granaries and labour constraints Land appropriation was a concern for all	Significant vulnerability (poverty, main economic activities are agriculture and pastoralism); 39% households are food insecure Respondents perceived themselves as less vulnerable to climate change because they already have adaptive capacity	A -No discussion of limitations, little reflexivity, ethics not acknowledged -Did not frame as climate change research to participants -Data saturation reached -Comprehensive methodology, data triangulation -Survey instrument was pre-tested -Findings were validated in feedback workshops
Ojala 2012 Sweden	Questionnaire with open-ended and Likert-type questions Qualitative analysis: thematic analysis Quantitative analysis: descriptive statistics of coded statements	To explore how young Swedish people cope with worry and promote hope in relation to climate change	*n* = 348 (M 127, F 221) Young people in Sweden Intermediate level school children: mean age 11.7 (*n* = 90) Senior high school adolescents: mean age 16.4 (*n* = 146) Young adult university students: mean age 22.6 (*n* = 112)	Problem-focused coping -individual (preparatory actions, direct actions) -collective Emotion-focused coping -de-emphasising seriousness of the problem (threat is exaggerated/not real, ego-centric thinking, relativisation) -distancing (distraction, avoidance) -social support -hyperactivation Meaning-focused coping -positive reappraisal (historical perspective) -positive thinking/existential hope -trust (in science/technology, politics and policy, business, environment movement, humanity, religion)		Age—young people most likely to experience impacts Location—stable Western country Some perceived climate change as not affecting them	A -Sampling poorly described -Little reflexivity -No evidence of ethics approval -Findings not validated -Aims clearly stated and met, high utility of research -Good contextualisation of background and results -Good response rate, large sample size and age range
Petheram et al. 2010 Australia	Fieldwork visits Semi-structured interviews (individual and group), workshops (rich picture diagramming, participatory sculpting, participant-generated photography) Constructionist/grounded theory-based continuous data gathering and analysis, analysis of data with substantive and theoretical coding	To understand factors influencing general vulnerability and adaptive capacity in the context of poverty and climate change in Yolgnu people in NE Arnhem Land, Northern Territory	*n* = 21 (M 9, F 12) Range of older and younger adults (ages not specified) Community members in East Arnhem Land (male indigenous land/sea rangers and women from local households and a women’s organisation)	Climate change was less of a concern than other issues affecting the community Climate change will exacerbate existing concerns, cannot be considered in isolation from non-climate issues Raising awareness of climate change so school children do not worry	Participants were unclear about Western notions of climate change Differences in world view and miscommunication between participants and “Balanda” (white Australians) Preferences for adaptation strategies included sustainability and greater value on traditional and cultural practices Specific changes in landscape had been noted in recent years and caused distress -Taking care of country -Concern about current and future situation of communities and wanting change to relieve poverty and other worrying issues	Population highly vulnerable to climate change (poverty, lack of agency and adaptive capacity, historic events, multiple pressing biopsychosocial issues) Indigenous population with strong connection to place and sensitivity to the natural landscape	A -No funding source nominated -Adapted research aims based on findings to be of more utility/relevance -Data saturation reached -Participant verification occurred -Used culturally adapted methods to gather data -Good reflexivity
Ryghaug et al. 2011 Norway	Focus group interviews Analysis ‘inspired’ by grounded theory; domestication theory used as a basis for making story lines	To analyse how people reason about and make sense of human-made global warming, in light of two previously identified categories of media representations, the “nature drama” and “science drama”	*n* = 62 in 10 focus groups (M 24, F 38) Age range 16–71 Community members in Norway Focus groups were existing social networks 26/62 (42%) were students	Climate change as a frightening scenario Worry for future generations was linked to extreme weather striking climate incidents (“nature drama”) Worry not pervasive, as frightening events are not happening here (their country) or now	Scientific controversy Role of the media as the main source of information about climate change, belief that media overemphasises threat Four ways of domesticating knowledge: acceptors, tempered acceptors, uncertain and sceptics	Participants from wealthy stable country Climate change perceived as distant from everyday concerns, less imminent than other problems	C -Bias in sampling -No comment on data saturation -No respondent validation -No discussion of limitations -No reflexivity, which is especially important given how subjects were recruited and inherent bias -Ethical flaws
Wright and Nyberg 2012 Australia	Individual semi-structured interviews and analysis of documents (strategy docs, communication material, submissions to gov’t, media coverage) Abductive approach Coding of emotional expressions using QSR NVivo software	(i) To explore how organisations have responded to the evidenced emotionality of climate change in their corporate environmental practices (ii) To explore how sustainability specialists manage their own emotions in the process of emotionology work	*n* = 36 (M 21, F 15) Age range 25–60 Sustainability specialists in corporate industries in Australia	“Climate change as threat” (anxiety and apprehension in regard to the future implications for society and the economy) Anxiety was harnessed to improve employee engagement, productivity, and corporate reputation Sustainability managers/consultants downplay threat and promote challenge/opportunity linked to business concerns Managing emotions through calculative methods, constraining, championing and compartmentalising emotions	“Climate change as battleground or conflict” (frustration, anger, and hostility) and “climate change as challenge and opportunity” (hope, enthusiasm, and excitement) Themes regarding processes of emotionology work (spanning, changing, or creating emotionologies)	Sustainability specialists working directly with climate change issues, all believed in climate change and were passionate Age, type of organisation and gender were not discussed as qualifiers	C -Poor presentation of data findings -No reflexivity -No comment on data saturation -Unclear how data from documents were gathered and analysed -Sampling method not described -No validation of findings -No dissenting views -Ethical flaws

## Data Availability

Not applicable.
